# Risk factors and prognosis of liver metastasis in gallbladder cancer patients: A SEER-based study

**DOI:** 10.3389/fsurg.2022.899896

**Published:** 2022-08-23

**Authors:** Cheng Fang, Wenhui Li, Qingqiang Wang, Ruoran Wang, Hui Dong, Junjie Chen, Yong Chen

**Affiliations:** ^1^ Department of Hepatic Surgery IV, Eastern Hepatobiliary Surgery Hospital, Second Military Medical University, Shanghai, China; ^2^ Department of Gynecology and Obstetrics, Changhai Hospital, Second Military Medical University, Shanghai, China; ^3^ Department of Hepatobiliary Surgery, Xijing Hospital, Fourth Military Medical University, Xian, China; ^4^ Department of Neurosurgery, West China Hospital, Sichuan University, Chengdu, China; ^5^ International Cooperation Laboratory on Signal Transduction, National Center for Liver Cancer, Eastern Hepatobiliary Surgery Hospital, Second Military Medical University, Shanghai, China; ^6^ Department of Ultrasonography, Eastern Hepatobiliary Surgery Hospital, Second Military Medical University, Shanghai, China

**Keywords:** gallbladder cancer, liver metastasis, risk factors, survival, nomogram

## Abstract

**Background:**

Liver metastasis is a common complication in gallbladder cancer (GBC). We design this study to develop models for predicting the development of liver metastasis in GBC patients and evaluate the risk of mortality in these patients with liver metastasis.

**Methods:**

GBC patients from Surveillance Epidemiology and End Results (SEER) between 2010 and 2016 were included in this study. Logistic regression was performed to discover risk factors and construct predictive models for liver metastasis in GBC patients. Cox regression was utilized to find risk factors of mortality in GBC patients with liver metastasis. The area under the receiver operating characteristic curve (AUC) was calculated to evaluate the performance of the constructed predictive models.

**Results:**

Multivariate logistic regression confirmed that T stage, N stage, and tumor grade were risk factors for liver metastasis in GBC patients. Composed of these factors, the model for predicting the development of liver metastasis had AUCs of 0.707 and 0.657 in the training cohort and testing cohort, respectively. Multivariate Cox regression showed that surgery of the primary site and chemotherapy were independently associated with the mortality of GBC patients with liver metastasis. Composed of these two factors, the predictive model for 1-year mortality of GBC patients with liver metastasis had AUCs of 0.734 and 0.776 in the training cohort and testing cohort, respectively.

**Conclusion:**

The predictive models that we constructed are helpful for surgeons to evaluate the risk of liver metastasis in GBC patients and the survival condition of those with liver metastasis. Surgery of the primary site and chemotherapy should be provided for GBC with liver metastasis.

## Introduction

With an incidence of 2.2 per 100 thousand annually, gallbladder cancer (GBC) accounts for 80%–95% of biliary system cancers around the world (1, 2). Although development in clinical practice including diagnosis and therapeutics has been achieved, the prognosis of GBC is still poor, with 5-year survival rates of 32.4% and 3.5% in the T3 and T4 stages, respectively (3). This fact may mainly be attributable to the contradiction between the high invasiveness and difficult identification of GBC for lacking obvious symptoms in the early phase (4). Therefore, GBC patients commonly develop distant metastasis in the initial clinic visit.

Previous population-based studies investigated that metastasis of lymph nodes and distant organs is prevalent in GBC, with incidence ranging from 17.9% to 64.5% (5, 6). The most frequently observed metastatic organs are the liver, lung, and peritoneum (7). The prognosis of metastatic GBC is poorer than those without metastasis, with 1-year survival rate ranging from 20% to 50% (6, 8, 9). Previous research studies showed that the liver is the leading metastasis, which is found in more than half of metastatic GBC (6, 7). Also, studies have confirmed liver metastasis as a significant risk factor for survival in GBC (6). Therefore, evaluating the risk of liver metastasis in GBC as early as possible is essential for clinicians to adopt preventive therapeutic options to improve the prognosis of GBC. However, there is still no study exploring the risk factors of liver metastasis in GBC. We design this study to explore the risk factors and develop predictive models for liver metastasis in GBC patients. In addition, we evaluate the risk of poor prognosis in GBC patients with liver metastasis in this study.

## Materials and methods

### Study population

**Figure 1:**
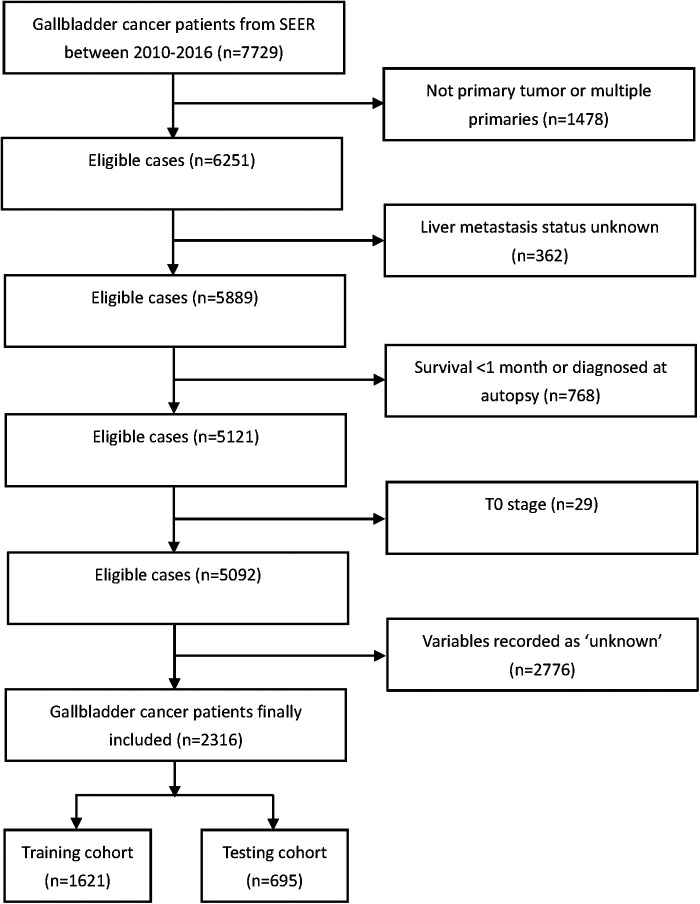
Inclusion flowchart of gallbladder cancer patients from the SEER database.

GBC patients in the Surveillance Epidemiology and End Results (SEER) database between 2010 and 2016 were extracted for this study using SEER Stat software version 8.3.9. The SEER database consists of 18 population-based cancer registries in 14 states of the United States and covers 28% of the US population. Patients were excluded if they met the following criteria: (1) no primary tumor or multiple primaries; (2) liver metastasis status unknown; (3) survival <1 month or diagnosed at autopsy; (4) T0 stage; and (5) variables recorded as “unknown.” The complete flowchart of patients’ inclusion is shown in Figure 1. Finally, 2,316 patients with GBC were included in the study. Data collected in the SEER are deidentified and are freely available to the public so that ethic approval from the institutional review board and written consent are not necessary for this study.

### Variable collection

Demographic variables including age at diagnosis, sex, insurance status (insured, uninsured), marital status (married, unmarried), race (white, black, and others), and tumor-related variables including histological type (adenocarcinoma, others), differentiated grade (I, II, III, IV), primary tumor size (<1 cm, 1–3 cm, 3–5 cm, ≥5 cm), T stage (T1, T2, T3, T4), and N stage (N0, N1, N2) were collected. Information about other metastatic locations including the lung, bone, and brain was also extracted as variables. Therapeutic options including surgery of primary lesions, lymphadenectomy, radiotherapy, and chemotherapy were included. The survival time and vital status were collected to define the outcomes. The outcomes of this study were the development of liver metastasis and 1-year overall mortality.

### Statisical analysis

Age was shown as a continuous variable in the form of median (quartile). Other categorical variables were presented as numbers (percentage). Patients were divided into two groups based on the development of liver metastasis. The differences in the categorical variables between the two groups were compared using the chi-square test or Fisher's exact test.

To construct models for predicting liver metastasis in GBC patients, the patients were randomly split into a training cohort and a testing cohort in a ratio of 7:3. Univariate logistic regression was first performed in the training cohort to discover the potential risk factors of liver metastasis. Then, factors with *p* < 0.05 in univariate logistic regression were included in multivariate logistic regression for analysis. Independent risk factors with *p* < 0.05 in multivariate logistic regression were incorporated to develop the model for predicting liver metastasis. The nomogram of the predictive model was drawn for convenient clinical use. The performance of the model was verified in the training cohort and testing cohort by calculating the area under the receiver operating characteristic curve (ROC) (AUC). A calibration plot was also drawn to assess the consistency between the actual probability and the predicted probability of liver metastasis.

The model for predicting 1-year overall mortality of GBC patients with liver metastasis was constructed using Cox regression. Univariate and multivariate Cox regression analyses were sequentially performed to discover risk factors for 1-year mortality in the training cohort. The nomogram of the Cox regression-based predictive model was drawn to evaluate the risk of outcomes visually. Both the ROC and calibration plot were drawn in the training cohort and testing cohort to evaluate the predictive accuracy and consistency.

R (version 3.6.1, R Foundation) and SPSS 23.0 Windows software (SPSS Inc., Chicago, IL) were implemented for statistical analyses and figures. A two-sided *p* value < 0.05 was defined as statistically significant.

## Results

### Baseline characteristics of included GBC patients

A total of 2,316 GBC patients were finally included in this study, with a liver metastasis incidence of 13.9% (Table 1). Patients with liver metastasis had lower age than those without liver metastasis (*p* = 0.015). Adenocarcinoma type was more common in patients without liver metastasis than those with liver metastasis (*p* < 0.001). Compared with patients without liver metastasis, those with liver metastasis had a higher tumor grade (*p* < 0.001) and tumor size (*p* < 0.001). Also, patients with liver metastasis had a higher T stage (*p* < 0.001) and N stage (*p* < 0.001) than those without liver metastasis. With regard to other metastatic locations, metastases of the lung (*p* < 0.001) and bone (*p* < 0.001) but not the brain (*p* = 1.000) were more frequently observed in patients with liver metastasis. The records of therapeutics showed that patients without liver metastasis had a higher incidence of surgery of the primary site (*p* < 0.001), lymphadenectomy (*p* < 0.001), and radiotherapy (*p* < 0.001) but a lower incidence of chemotherapy (*p* < 0.001). Finally, the 1-year mortality of overall patients was 36.7%, and the mortality was significantly higher in those with liver metastasis (*p* < 0.001).

**Table 1:** Baseline characteristics of included gallbladder cancer patients.

Variables	All patients (*n* = 2,316)	Patients without liver metastasis (*n* = 1,995, 86.1%)	Patients with liver metastasis (*n* = 321, 13.9%)	*p*
Age	69 (59–76)	69 (60–78)	69 (60–78)	**0.015**
Male gender	679 (29.3)	591 (29.6)	88 (27.4)	0.459
Race				0.200
White	1,705 (73.6)	1,468 (73.6)	237 (73.8)	
Black	308 (13.3)	258 (12.9)	50 (15.6)	
Others	303 (13.1)	269 (13.5)	34 (10.6)	
Insurance status				0.304
Insured	2,202 (95.1)	1,901 (95.3)	301 (93.8)	
Uninsured	114 (4.9)	94 (4.7)	20 (6.2)	
Marital status				0.659
Married	1,182 (51.0)	1,014 (50.8)	168 (52.3)	
Unmarried	1,134 (49.0)	981 (49.2)	153 (47.7)	
Histological type				**<0.001**
Adenocarcinoma	2,017 (87.1)	1,760 (88.2)	257 (80.1)	
Others	299 (12.9)	235 (11.8)	64 (19.9)	
Grade				**<0.001**
I	326 (14.1)	310 (15.5)	16 (5.0)	
II	1,017 (43.9)	899 (45.1)	118 (36.8)	
III	914 (39.5)	743 (37.2)	171 (53.3)	
IV	59 (2.5)	43 (2.2)	16 (5.0)	
Tumor size				**<0.001**
<1 cm	158 (6.8)	152 (7.6)	6 (1.9)	
1–3 cm	890 (38.4)	794 (39.8)	96 (29.9)	
3–5 cm	622 (26.9)	526 (26.4)	96 (29.9)	
≥5 cm	646 (27.9)	523 (26.2)	123 (38.3)	
T stage				**<0.001**
T1	317 (13.7)	302 (15.1)	15 (4.7)	
T2	1,006 (43.4)	917 (46.0)	89 (27.7)	
T3	921 (39.8)	716 (35.9)	205 (63.9)	
T4	72 (3.1)	60 (3.0)	12 (3.7)	
N stage				**<0.001**
N0	1,540 (66.5)	1,382 (69.3)	158 (49.2)	
N1	668 (28.8)	540 (27.1)	128 (39.9)	
N2	108 (4.7)	73 (3.7)	35 (10.9)	
Lung metastasis				**<0.001**
Yes	44 (1.9)	22 (1.1)	22 (6.9)	
No	2,272 (98.1)	1,973 (98.9)	299 (93.1)	
Bone metastasis				**<0.001**
Yes	25 (1.1)	15 (0.8)	10 (3.1)	
No	2,291 (98.9)	1,980 (99.2)	311 (96.9)	
Brain metastasis				1.000
Yes	4 (0.2)	3 (0.2)	1 (0.3)	
No	2,312 (99.8)	1,992 (99.8)	320 (99.7)	
Surgery of the primary site				**<0.001**
Yes	2,151 (92.9)	1,903 (95.4)	248 (77.3)	
No	165 (7.1)	92 (4.6)	73 (22.7)	
Lymphadenectomy				**<0.001**
Yes	1,219 (52.6)	1,102 (55.2)	117 (36.4)	
No	1,097 (47.4)	893 (44.8)	204 (63.6)	
Radiotherapy				**<0.001**
Yes	432 (18.7)	400 (20.1)	32 (10.0)	
No	1,884 (81.3)	1,595 (79.9)	289 (90.0)	
Chemotherapy				**<0.001**
Yes	999 (43.1)	822 (41.2)	177 (55.1)	
No	1,317 (56.9)	1,173 (58.8)	144 (44.9)	
1-year mortality	850 (36.7)	630 (31.6)	220 (68.5)	**<0.001**

Bold value indicated *p* < 0.05.

### Risk factors of liver metastasis in GBC patients

**Figure 2:**
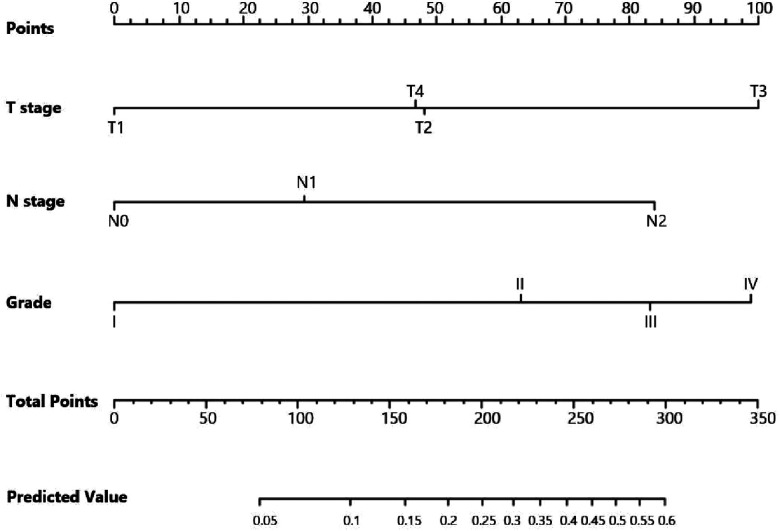
Nomogram for predicting liver metastasis in gallbladder cancer patients.

**Figure 3:**
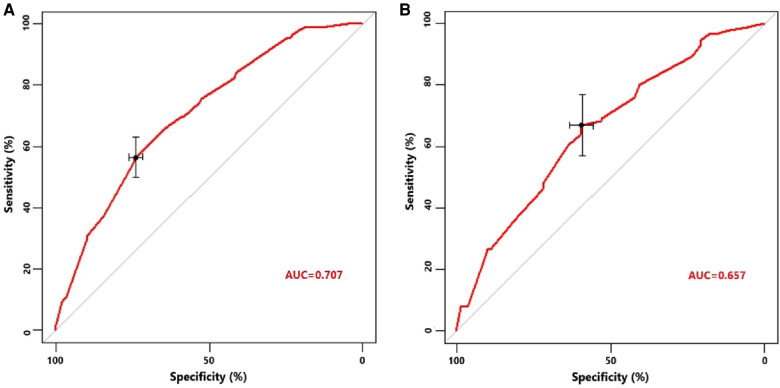
(**A**) Receiver operating characteristic curve of the constructed nomogram for predicting liver metastasis in the training cohort. (**B)** Receiver operating characteristic curve of the constructed nomogram for predicting liver metastasis in the testing cohort.

**Figure 4:**
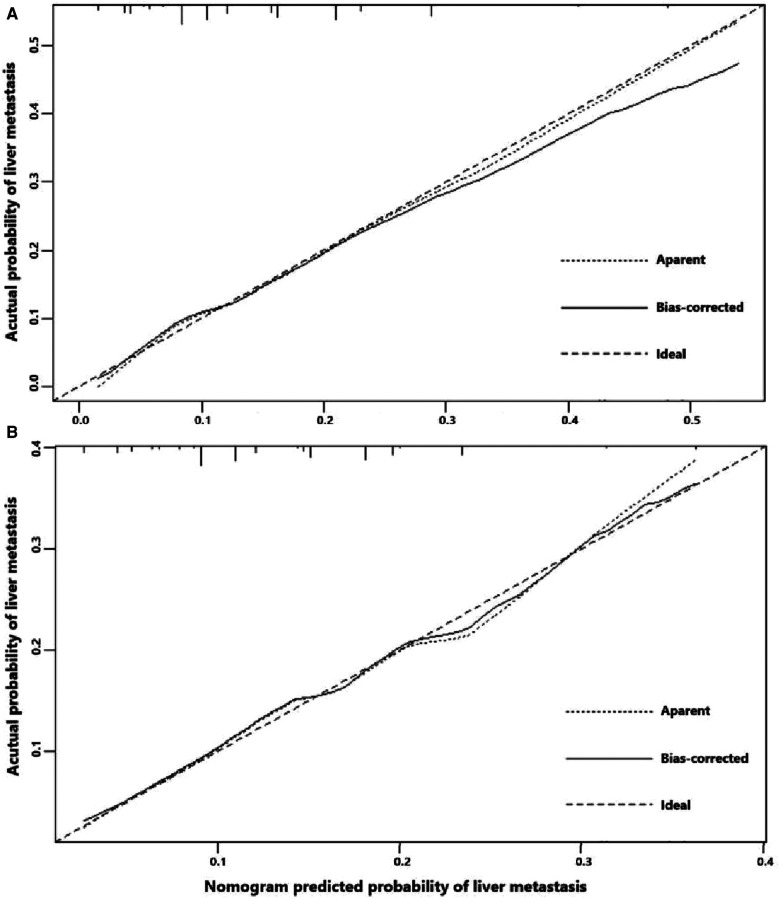
(**A**) Calibration curve of the constructed nomogram for predicting liver metastasis in the training cohort. (**B**) Calibration curve of the constructed nomogram for predicting liver metastasis in the testing cohort.

Univariate logistic regression showed that age (*p* = 0.034), histological type (*p* = 0.010), grade (<0.001), tumor size (<0.001), T stage (*p* < 0.001), and N stage (*p* < 0.001) were all significantly associated with the development of liver metastasis in GBC patients (Table 2). While after adjusting for confounding effects, multivariate logistic regression confirmed that grade (*p* = 0.006), T stage (*p* < 0.001), and N stage (*p* < 0.001) were independent risk factors of liver metastasis. Significant factors in multivariate logistic regression were combined to construct a predictive model for liver metastasis. The predictive model was visually presented as the nomogram in Figure 2. The ROC curves of the nomogram for predicting liver metastasis in the training cohort and testing cohort are shown in Figures 3A,B. The AUCs of the predictive nomogram in the training cohort and testing cohort were 0.707 (95% CI: 0.673–0.742) and 0.657 (95% CI: 0.599–0.716), respectively (Table 3). Calibration curves of the nomogram in the training cohort and testing cohort are shown in Figures 4A,B.

**Table 2:** Risk factors for liver metastasis in gallbladder cancer patients analyzed by logistic regression.

Variables	Univariate logistic regression			Multivariate logistic regression		
	OR	95% CI	*p*	OR	95% CI	*p*
Age	0.988	0.977–0.999	**0.034**	0.988	0.977–1.000	0.056
Male gender	0.908	0.666–1.236	0.539			
Race			0.371			
White	1.000	[Reference]				
Black	1.082	0.722–1.621	0.702			
Others	0.738	0.465–1.171	0.197			
Insurance status			0.414			
Insured	1.000	[Reference]				
Uninsured	0.781	0.431–1.414	0.414			
Marital status			0.321			
Married	1.000	[Reference]				
Unmarried	1.152	0.871–1.525	0.321			
Histological type			**0.010**			0.679
Adenocarcinoma	1.000	[Reference]		1.000	[Reference]	
Others	0.618	0.428–0.892	**0.010**	1.088	0.729–1.626	0.679
Grade			**<0.001**			**0** **.** **006**
I	1.000	[Reference]		1.000	[Reference]	
II	3.647	1.738–7.652	**0.001**	2.539	1.195–5.396	**0** **.** **015**
III	6.230	2.994–12.960	**<0.001**	3.391	1.597–7.203	**0** **.** **001**
IV	9.591	3.647–25.222	**<0.001**	4.261	1.530–11.872	**0** **.** **006**
Tumor size			**<0.001**			0.590
< 1 cm	1.000	[Reference]		1.000	[Reference]	
1–3 cm	3.647	1.738–7.652	**0.001**	1.951	0.676–5.628	0.216
3–5 cm	6.230	2.994–12.960	**<0.001**	2.053	0.703–5.994	0.188
≥5 cm	9.591	3.647–25.222	**<0.001**	2.114	0.722–6.189	0.172
T stage			**<0.001**			**<0.001**
T1	1.000	[Reference]		1.000	[Reference]	
T2	2.598	1.275–5.295	**0.009**	1.959	0.942–4.070	0.072
T3	7.179	3.595–14.336	**<0.001**	4.093	1.978–8.468	**<0.001**
T4	3.380	1.144–9.988	**0.028**	1.783	0.573–5.547	0.318
N stage			**<0.001**			**<0.001**
N0	1.000	[Reference]		1.000	[Reference]	
N1	2.059	1.523–2.783	**<0.001**	1.507	1.099–2.067	**0** **.** **011**
N2	5.401	3.214–9.077	**<0.001**	3.382	1.957–5.843	**<0.001**

Bold values indicated *p* < 0.05.

**Table 3:** Performance of a constructed nomogram for predicting liver metastasis in the training cohort and testing cohort.

	AUC	95% CI	Sensitivity	Specificity
Training cohort	0.707	0.673–0.742	0.565	0.740
Testing cohort	0.657	0.599–0.716	0.670	0.594

AUC, area under the receiver operating characteristic curve; CI, confidence interval.

### Prognosis of GBC patients with liver metastasis

**Figure 5:**
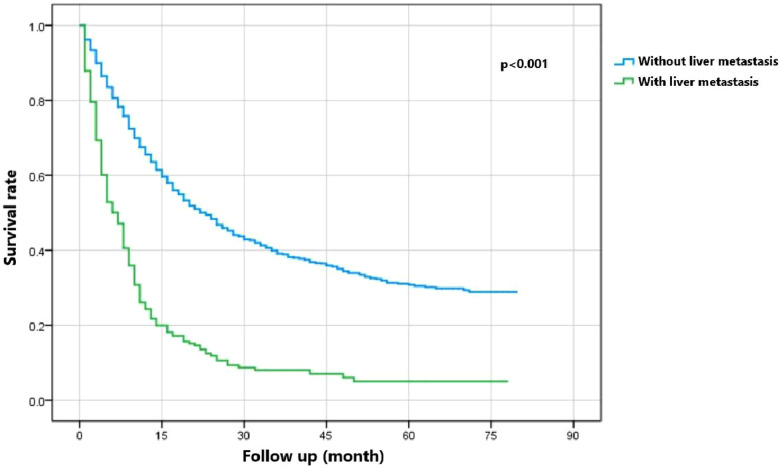
Survival curve of gallbladder cancer patients with and without liver metastasis.

**Figure 6:**
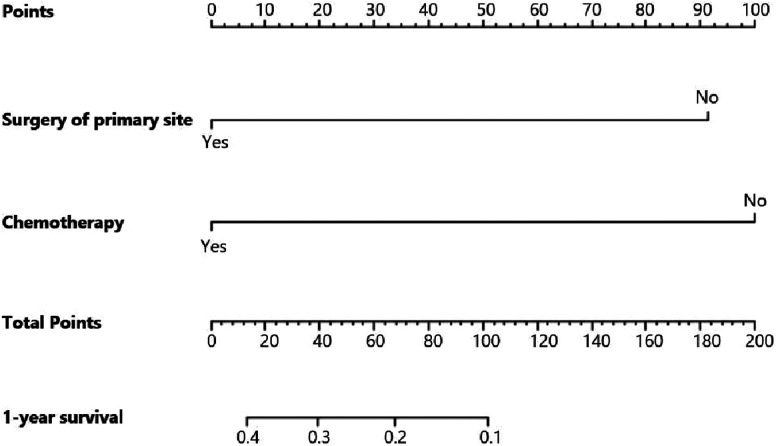
Nomogram for predicting 1-year survival of gallbladder cancer patients with liver metastasis.

**Figure 7:**
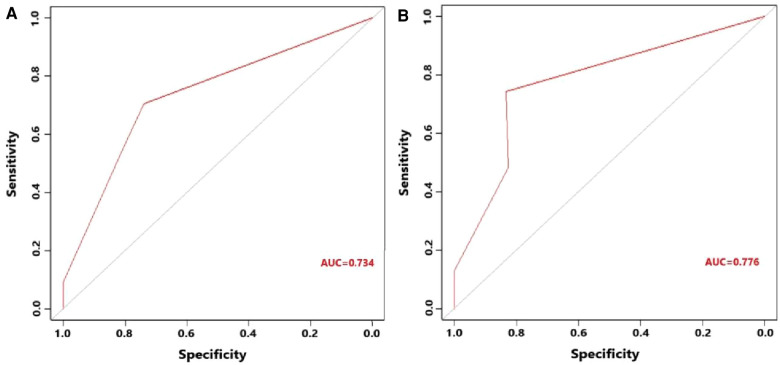
(**A**) Receiver operating characteristic curve of the constructed nomogram for predicting 1-year survival of gallbladder cancer patients with liver metastasis in the training cohort. (**B**) Receiver operating characteristic curve of the constructed nomogram for predicting 1-year survival of gallbladder cancer patients with liver metastasis in the testing cohort.

**Figure 8:**
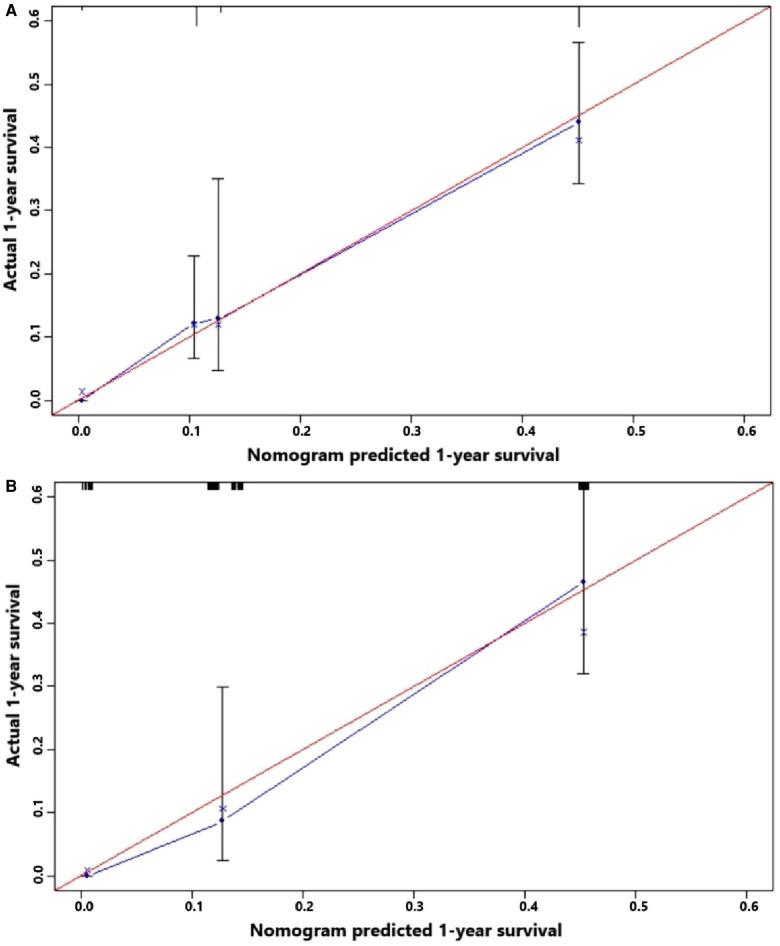
(**A**) Calibration curve of the constructed nomogram for predicting 3-year survival of gallbladder cancer patients with liver metastasis in the training cohort. (**B**) Calibration curve of the constructed nomogram for predicting 3-year survival of gallbladder cancer patients with liver metastasis in the testing cohort.

Figure 5 indicates that patients with liver metastasis had a significantly lower 1-year survival rate than those without liver metastasis (*p* < 0.001). The median survival of GBC patients with and without liver metastasis was 7 and 22 months, respectively. Univariate Cox regression indicated that T stage (*p* = 0.043), surgery of the primary site (*p* < 0.001), lymphadenectomy (*p* < 0.001), radiotherapy (*p* = 0.003), and chemotherapy (*p* < 0.001) were potential risk factors of mortality in GBC patients with liver metastasis (Table 4). However, after adjusting for confounding effects, multivariate Cox regression presented that only surgery of the primary site (*p* = 0.001) and chemotherapy (*p* < 0.001) were independently associated with mortality of GBC patients with liver metastasis. Composed of surgery of the primary site and chemotherapy, the predictive model for 1-year mortality of GBC patients with liver metastasis is visually shown as a nomogram in Figure 6. The ROC curves of the nomogram for predicting 1-year mortality in the training cohort and testing cohort are presented in Figures 7A,B. The AUCs of the predictive nomogram in the training cohort and testing cohort were 0.734 (95% CI: 0.700–0.769) and 0.776 (95% CI: 0.718–0.835), respectively (Table 5). Calibration curves of the nomogram in the training cohort and testing cohort are presented in Figures 8A,B, respectively.

**Table 4:** Risk factors for prognosis of gallbladder cancer patients with liver metastasis analyzed by Cox regression.

Variables	Univariate Cox regression			Multivariate Cox regression		
	HR	95% CI	*p*	HR	95% CI	*p*
Age	1.010	0.997–1.022	0.132			
Male gender	1.043	0.756–1.437	0.799			
Race			0.949			
White	1.000	[Reference]				
Black	0.954	0.635–1.432	0.820			
Others	0.937	0.573–1.531	0.795			
Insurance status						
Insured	1.000	[Reference]				
Uninsured	1.559	0.844–2.877	0.156			
Marital status						
Married	1.000	[Reference]				
Unmarried	1.144	0.855–1.530	0.366			
Histological type			0.142			
Adenocarcinoma	1.000	[Reference]				
Others	1.315	0.913–1.894	0.142			
Grade			0.139			
I	1.000	[Reference]				
II	0.866	0.397–1.889	0.718			
III	1.216	0.565–2.618	0.618			
IV	1.431	0.551–3.715	0.461			
Tumor size			0.245			
<1 cm	1.000	[Reference]				
1–3 cm	0.601	0.255–1.420	0.246			
3–5 cm	0.990	0.693–1.413	0.954			
≥5 cm	1.305	0.913–1.866	0.144			
T stage			**0.043**			0.862
T1	1.000	[Reference]		1.000	[Reference]	
T2	1.653	0.964–2.835	0.068	0.585	1.788–1.023	0.937
T3	0.679	0.485–0.951	0.024	0.598	1.242–0.862	0.424
T4	0.991	0.734–1.338	0.954	0.678	1.243–0.918	0.579
N stage			0.702			
N0	1.000	[Reference]				
N1	0.914	0.740–1.129	0.403			
N2	0.993	0.800–1.234	0.952			
Lung metastasis			0.134			
No	1.000	[Reference]				
Yes	1.500	0.883–2.549	0.134			
Bone metastasis			0.246			
No	1.000	[Reference]				
Yes	1.569	0.734–3.354	0.246			
Brain metastasis			0.279			
No	1.000	[Reference]				
Yes	2.976	0.413–21.434	0.279			
Surgery of the primary site			**<0.001**			**0.001**
No	1.000	[Reference]		1.000	[Reference]	
Yes	0.497	0.352–0.702	**<0.001**	0.326	0.751–0.495	**0.001**
Lymphadenectomy			**<0.001**			0.103
No	1.000	[Reference]		1.000	[Reference]	
Yes	0.557	0.410–0.756	**<0.001**	0.535	1.059–0.752	0.103
Radiotherapy			**0.003**			0.148
No	1.000	[Reference]		1.000	[Reference]	
Yes	0.471	0.288–0.771	**0.003**	0.400	1.148–0.678	0.148
Chemotherapy			**<0.001**			**<0.001**
No	1.000	[Reference]		1.000	[Reference]	
Yes	0.418	0.311–0.562	**<0.001**	0.308	0.592–0.427	**<0.001**

Bold values indicated *p* < 0.05.

**Table 5:** Performance of a constructed nomogram for predicting 1-year survival of gallbladder cancer patients with liver metastasis in the training and testing cohorts.

	AUC	95% CI	Sensitivity	Specificity
Training cohort	0.734	0.700–0.769	0.704	0.740
Testing cohort	0.776	0.718–0.835	0.744	0.833

AUC, area under the receiver operating characteristic curve; CI, confidence interval.

## Discussion

Although GBC is a rare cancer of the gastrointestinal system, the prognosis of GBC, especially T3 and T4, is poor. Previous studies have shown that the 1-year and 3-year cancer-specific survival rates of non-metastatic GBC were 82.3% and 56.7%, respectively (10). The GBC with metastasis had a shorter survival time than non-metastatic GBC and was commonly observed in the advanced stage and non-adenocarcinoma type (5). The three major metastatic pathways of GBC include hematogenous metastasis, lymphatic metastasis, and direct infiltration. More than half of the GBC could directly infiltrate into adjacent organs such as the liver, bile duct, pancreas, stomach, duodenum, and omentum (11). Due to the adjacency between the liver and the gallbladder, the liver is the leading metastatic site accounting for more than 50% of metastatic GBC (6, 12). Because liver metastasis would obviously shorten the survival time of GBC, it is very important to evaluate the risk of liver metastasis in the early stage and formulate preventive measures and adjuvant treatments. The AUCs of our developed model for predicting liver metastasis in the training cohort and testing cohort are 0.707 and 0.657, respectively, which indicates that this model is helpful for surgeons to evaluate the risk with relatively high accuracy.

Three factors were finally included in the developed nomogram for predicting liver metastasis, namely, histological grade, T stage, and N stage. The higher histological grade was confirmed to be positively associated with a higher likelihood of liver metastasis in GBC. Actually, a higher histological grade is commonly characterized as higher invasiveness and a wider infiltration range, leading to a higher risk of metastasis. One previous study also observed that poorly differentiated GBC was more probably to develop disseminated disease including peritoneal disease, liver metastasis, and retroperitoneal disease (13). Additionally, T2, T3, and T4 and N2, and N1 were verified to have a higher risk of liver metastasis than T1 and N0 in our study. The micrometastasis of regional lymph nodes undoubtedly indicates more aggressive biological behavior of GBC and sequentially a higher possibility of distant metastasis and poor prognosis in GBC patients (14).

Because a large number of GBC patients had developed liver metastasis during the first clinical visit, developing a suitable therapeutic plan is essential to improve the prognosis of these metastatic GBC patients. Also, our study confirmed that the median survival of GBC with liver metastasis was significantly shorter than those without liver metastasis. Therefore, evaluating the risk of poor prognosis in GBC with liver metastasis is essential for surgeons to select optimal therapeutic options for these patients. The nomogram that we constructed for predicting the 1-year survival of those with liver metastasis showed a relatively good performance with AUCs of 0.734 and 0.776 in the training cohort and testing cohort, respectively. Two factors were incorporated into this nomogram: surgery of the primary site and chemotherapy.

In our study, patients receiving surgery of the primary site or chemotherapy had a higher possibility of alive status. There is still no widely acknowledged standard therapeutics for metastatic GBC patients, although both the European Society of Medical Oncology (ESMO) and the 2020 National Comprehensive Cancer Network (NCCN) recommended the use of chemotherapy for metastatic GBC. However, one previous pooled analysis of clinical trials conducted in 2006 showed that the effect of systemic chemotherapy was unsatisfactory, with a response rate of 22.6% and a tumor control rate of 57.3% (15). Recently, several clinical trials have been conducted to evaluate the effect of chemotherapy on biliary tract cancer. Including 447 patients with biliary tract malignancies after surgical resection, the BILCAP (biliary capecitabine) randomized controlled phase III study indicated that adjuvant capecitabine could improve survival rates compared with placebo (16). Another phase II trial showed that the combination of gemcitabine and capecitabine plus radiotherapy for extrahepatic cholangiocarcinoma and gallbladder carcinoma contributed to a 2-year survival of 65% and a median survival of 35 months (17). One recent randomized phase II study explored the effect of adding ramucirumab or merestinib to the standard cisplatin–gemcitabine chemotherapy but found no improved progression-free survival in patients with locally advanced or metastatic biliary tract cancer (18). Whatever, postoperative chemoradiotherapy should be provided for metastatic GBC including those with liver metastasis, while the optimal chemotherapy strategies and drugs should be investigated in future randomized trials. The importance of surgery of the primary site on the nomogram indicted that cholecystectomy should be provided for GBC with liver metastasis. A SEER-based study also verified that chemotherapy plus resection of the primary tumor was superior to chemotherapy alone in GBC with isolated liver metastasis (19). Also, one recent study confirmed that R0 resection with adjuvant chemotherapy was beneficial for the long-term survival of stage III or IV GBC patients (20). Another study focusing on gallbladder adenocarcinoma showed surgery of the primary site was independently associated with the longer survival of metastatic gallbladder adenocarcinoma patients, although it was less used in these patients (6). Finally, radiotherapy was not confirmed as an important factor influencing the survival of GBC with liver metastasis, which was consistent with the results of previous studies (6). To sum up, the nomogram that we constructed incorporating surgery of the primary site and chemotherapy could not only evaluate the risk of 1-year survival of GBC with liver metastasis but also emphasize the importance of cholecystectomy and developing a novel chemotherapy regimen for metastatic GBC patients.

This study has several limitations. First, although this study collected data from the SEER database with a large sample size, the deficiency of a retrospective study such as selection bias could not be avoided. Second, the information provided in the SEER database is relatively simplistic. Many variables, including comorbidities, perineural invasion, and metastasis of peritoneum and adjacent organs, which may affect the risk of liver metastasis and survival, are not recorded in the SEER database. Finally, although all patients were divided into the training cohort and testing cohort for internal validation of developed models, external validation in other regions such as East Asia and South America with a high incidence of GBC remains worthwhile to be performed by future studies.

## Conclusion

The predictive nomogram that we constructed is efficient for surgeons to predict the liver metastasis in GBC and the survival status of those with liver metastasis. Surgery of the primary site and chemotherapy should be provided for GBC patients with liver metastasis.

## Data Availability

The raw data supporting the conclusions of this article will be made available by the authors without undue reservation.

## References

[1] Ertel AE Bentrem D Abbott DE. Gallbladder cancer. Cancer Treat Res. (2016) 168:101–20. 10.1007/978-3-319-34244-3_6. 29206367

[2] Ferlay J Soerjomataram I Dikshit R Eser S Mathers C Rebelo M Cancer incidence and mortality worldwide: sources, methods and major patterns in GLOBOCAN 2012. Int J Cancer. (2015) 136(5):E359–86. 10.1002/ijc.29210. 25220842

[3] Lim H Seo DW Park DH Lee SS Lee SK Kim MH Prognostic factors in patients with gallbladder cancer after surgical resection: analysis of 279 operated patients. J Clin Gastroenterol. (2013) 47(5):443–8. 10.1097/MCG.0b013e3182703409. 23188077

[4] Baiu I Visser B. Gallbladder cancer. JAMA (2018) 320(12):1294. 10.1001/jama.2018.11815. 30264121

[5] Cai YL Lin YX Jiang LS Ye H Li FY Cheng NS. A novel nomogram predicting distant metastasis in T1 and T2 gallbladder cancer: a SEER-based study. Int J Med Sci. (2020) 17(12):1704–12. 10.7150/ijms.47073. 32714073 PMC7378661

[6] Yang Y Tu Z Ye C Cai H Yang S Chen X Site-specific metastases of gallbladder adenocarcinoma and their prognostic value for survival: a SEER-based study. BMC Surg. (2021) 21(1):59. 10.1186/s12893-021-01068-8. 33485332 PMC7825172

[7] Wang X Yu GY Chen M Wei R Chen J Wang Z. Pattern of distant metastases in primary extrahepatic bile-duct cancer: a SEER-based study. Cancer Med. (2018) 7(10):5006–14. 10.1002/cam4.1772. 30277653 PMC6198228

[8] Mady M Prasai K Tella SH Yadav S Hallemeier CL Rakshit S Neutrophil to lymphocyte ratio as a prognostic marker in metastatic gallbladder cancer. HPB (2020) 22(10):1490–5. 10.1016/j.hpb.2020.02.002. 32122786

[9] Du JH Lu J. Circulating CEA-dNLR score predicts clinical outcome of metastatic gallbladder cancer patient. J Clin Lab Anal. (2019) 33(2):e22684. 10.1002/jcla.22684. 30461064 PMC6818570

[10] Zhang W Hong HJ Chen YL. Establishment of a gallbladder cancer-specific survival model to predict prognosis in non-metastatic gallbladder cancer patients after surgical resection. Dig Dis Sci. (2018) 63(9):2251–8. 10.1007/s10620-018-5103-7. 29736837

[11] McNamara MG Metran-Nascente C Knox JJ. State-of-the-art in the management of locally advanced and metastatic gallbladder cancer. Curr Opin Oncol. (2013) 25(4):425–31. 10.1097/CCO.0b013e3283620fd8. 23635800

[12] Wang J Bo X Nan L Wang CC Gao Z Suo T Landscape of distant metastasis mode and current chemotherapy efficacy of the advanced biliary tract cancer in the United States, 2010–2016. Cancer Med. (2020) 9(4):1335–48. 10.1002/cam4.2794. 31876990 PMC7013071

[13] Butte JM Gönen M Allen PJ D'Angelica MI Kingham TP Fong Y The role of laparoscopic staging in patients with incidental gallbladder cancer. HPB (2011) 13(7):463–72. 10.1111/j.1477-2574.2011.00325.x. 21689230 PMC3133713

[14] Nagakura S Shirai Y Yokoyama N Hatakeyama K. Clinical significance of lymph node micrometastasis in gallbladder carcinoma. Surgery (2001) 129(6):704–13. 10.1067/msy.2001.114764. 11391369

[15] Eckel F Schmid RM. Chemotherapy in advanced biliary tract carcinoma: a pooled analysis of clinical trials. Br J Cancer. (2007) 96(6):896–902. 10.1038/sj.bjc.6603648. 17325704 PMC2360111

[16] Primrose JN Fox RP Palmer DH Malik HZ Prasad R Mirza D Capecitabine compared with observation in resected biliary tract cancer (BILCAP): a randomised, controlled, multicentre, phase 3 study. Lancet Oncol. (2019) 20(5):663–73. 10.1016/s1470-2045(18)30915-x. 30922733

[17] Ben-Josef E Guthrie KA El-Khoueiry AB Corless CL Zalupski MM Lowy AM SWOG S0809: a phase II intergroup trial of adjuvant capecitabine and gemcitabine followed by radiotherapy and concurrent capecitabine in extrahepatic cholangiocarcinoma and gallbladder carcinoma. J Clin Oncol. (2015) 33(24):2617–22. 10.1200/jco.2014.60.2219 25964250 PMC4534524

[18] Valle JW Vogel A Denlinger CS He AR Bai LY Orlova R Addition of ramucirumab or merestinib to standard first-line chemotherapy for locally advanced or metastatic biliary tract cancer: a randomised, double-blind, multicentre, phase 2 study. Lancet Oncol. (2021) 22(10):1468–82. 10.1016/s1470-2045(21)00409-5. 34592180

[19] Zhu J Hu W Zhang Y Du P Xiao W Li Y. Comparison of survival outcomes of chemotherapy plus surgery vs chemotherapy alone for patients with isolated liver metastases from gallbladder carcinoma. Am Surg. (2021):31348211038563. 10.1177/00031348211038563. [Epub ahead of print] 34382879

[20] Yuza K Sakata J Hirose Y Miura K Ando T Katada T Outcome of radical surgery for gallbladder carcinoma according to TNM stage: implications for adjuvant therapeutic strategies. Langenbeck's Arch Surg. (2021) 406(3):801–11. 10.1007/s00423-020-02068-7. 33398448

